# Atrial Fibrillation Treatment Stratification Based on Artificial Intelligence‐Driven Analysis of the Electrophysiological Complexity

**DOI:** 10.1111/jce.16754

**Published:** 2025-06-04

**Authors:** Ana María Sánchez de la Nava, Santiago Ros, Alejandro Carta, Esteban González‐Torrecilla, Ana González Mansilla, Javier Bermejo, Ángel Arenal, Andreu M. Climent, María S. Guillem, Felipe Atienza, Other Members of the STRATIFY Study Group

**Affiliations:** ^1^ Department of Cardiology Hospital General Universitario Gregorio Marañón, Instituto de Investigación Sanitaria Gregorio Marañón Madrid Spain; ^2^ CIBERCV, Centro de Investigación Biomédica en Red de Enfermedades Cardiovasculares Spain; ^3^ ITACA Universitat Politècnica de València Valencia Spain; ^4^ Facultad de Medicina Universidad Complutense de Madrid Madrid Spain; ^5^ Corify Care SL Madrid Spain

**Keywords:** artificial intelligence, atrial fibrillation, ECGI, stratification

## Abstract

**Background:**

Atrial Fibrillation (AF) treatment strategies are suboptimal and clinical predictors of success are limited. Artificial Intelligence (AI) has arisen as a powerful tool for treatment efficacy prediction.

**Objective:**

We developed an AI‐driven platform for the stratification of patients based on noninvasive Electrocardiographic Imaging (ECGI) biomarkers and clinical parameters to evaluate and predict optimal patient treatment.

**Methods:**

We evaluated 204 patients treated according to clinical guidelines and characterized them at the electrophysiological level using ECGI recordings during AF. ECGI signals were calculated to obtain frequency and rotational biomarkers. Baseline clinical characteristics and treatment after inclusion were registered.

**Results:**

A clustering algorithm was calibrated taking three different variables for 1 year outcome prediction: (1) AF type (paroxysmal or persistent); (2) ECGI complexity score (calculated based on highest dominant frequency, median dominant frequency, and mean rotor time); and (3) type of treatment: rhythm control (drugs, AF ablation) or rate control. The cluster analysis classified patients into five groups: Low electrophysiological complexity patterns were associated with an improved outcome after ablation, regardless of the time duration of the AF. Intermediate complexity scores in paroxysmal AF had a favourable outcome with rhythm control treatments, but not in persistent AF patients. Cluster patterns with higher electrophysiological complexity were associated with a higher probability of AF recurrence, both in paroxysmal and persistent groups. The performance of the algorithm predicting the outcome was (AUC: 0.73 (0.63–0.81)), increasing overall performance with respect to conventional persistent and paroxysmal classification (AUC: 0.58 (0.48–0.68); *p* < 0.05). This algorithm was evaluated on the 20% test set, obtaining 90% prediction success.

**Conclusions:**

AI‐driven analysis that combined clinical information with ECGI biomarkers increased the performance of conventional classification methods for AF treatment stratification.

**Abbreviations**
AF: atrial fibrillation
AI: artificial intelligence
DF: dominant frequency
ECGI: electrocardiographic imaging
PVI: pulmonary vein isolation
SR: sinus rhythm

## 1 Introduction

Atrial fibrillation (AF) ablation therapy increases survival in selected subgroups of patients, and improves quality of life, at the cost of increased expenditures, procedural risks, and arrhythmia recurrence [1]. Therefore, stratification methods are critical to accurately select patients to undergo successful ablation procedures, which is restricted by clinical characteristics and limitations of ablation procedures' capability. Multiple observational studies have identified clinical predictors of arrhythmia recurrence, but they are suboptimal. Recent guidelines from the European Heart Rhythm Association, Heart Rhythm Society, Asia Pacific Heart Rhythm Society, and Latin American Heart Rhythm Society emphasize the importance of noninvasive mapping tools, such as Electrocardiographic Imaging (ECGI), for the stratification of AF patients [2]. ECGI offers a noninvasive, simultaneous, and global characterization of biatrial electrical activity, which is particularly valuable for identifying AF driving sources, enhancing the decision‐making process for treatment strategies and may potentially help in the design of the ablation procedure [3, 4, 5, 6, 7]. This technology, in combination with advanced computational calculations such as neural networks and score prediction in combination with clinical baseline characteristics, may improve the identification of new biomarkers to evaluate AF prognosis [8].

We hypothesized that the electrophysiological complexity evaluated using ECGI, in combination with clinical parameters, may help to predict long‐term treatment efficacy (freedom from AF at 1 year) of patients with AF. To that purpose, we analyzed AF complexity using ECGI and developed a new stratification score to identify and predict the most suitable treatment.

## 2 Methods

### 2.1 Data and Study Population

We prospectively included AF patients from two different cohorts: (1) ambulatory patients presenting AF at the outpatient clinic, and (2) patients submitted for AF ablation. We excluded patients enrolled in another investigational study, implanted device, and contraindications for AF ablation. Patients were treated according to the clinicians' choice according to the ESC guidelines [1, 2]. The type of treatment was classified in four different categories: ablation (pulmonary vein isolation (PVI) or PVI isolation plus driver ablation), pharmacologic rhythm control (including cardioversion), or frequency rate control (drug/devices implant) [1, 2, 9, 10]. Clinical follow‐up included Holter recordings at 6, 9, and 12 months and changes in treatment strategies (pharmacological, electrical cardioversion, and/or redo ablation). We collected clinical variables, including age, gender, and treatment type, together with ECGI‐based electrical parameters.

The protocol was approved by the Institutional Ethics Committee of the institution and was registered at https://clinicaltrials.gov/ as STRATIFY trial (NCT04578275). All patients gave informed consent.

### 2.2 ECGI Recordings

The Imageless ECGI methodology (ACORYS, Corify Care S.L., Spain) has been described in prior publications [3, 4, 9, 10]. Briefly, body surface signals were recorded from patients during AF episodes, including a total of 63 electrodes homogeneously distributed over the torso of the patient. Signals were recorded in the resting state in the case of the outpatient group or under anaesthesia in the ablation group at a sampling frequency of 1 kHz. The geometry of the torso was reconstructed by photogrammetry to identify the electrode location and the overall geometry of the patient's thorax [3, 4, 8, 9, 10]. Using a statistical approach based on the thorax shape of each patient, we estimated a 3D bi‐atrial geometry mesh, including location and rotation [3, 4, 10, 11].

### 2.3 Data Processing

**Figure 1:**
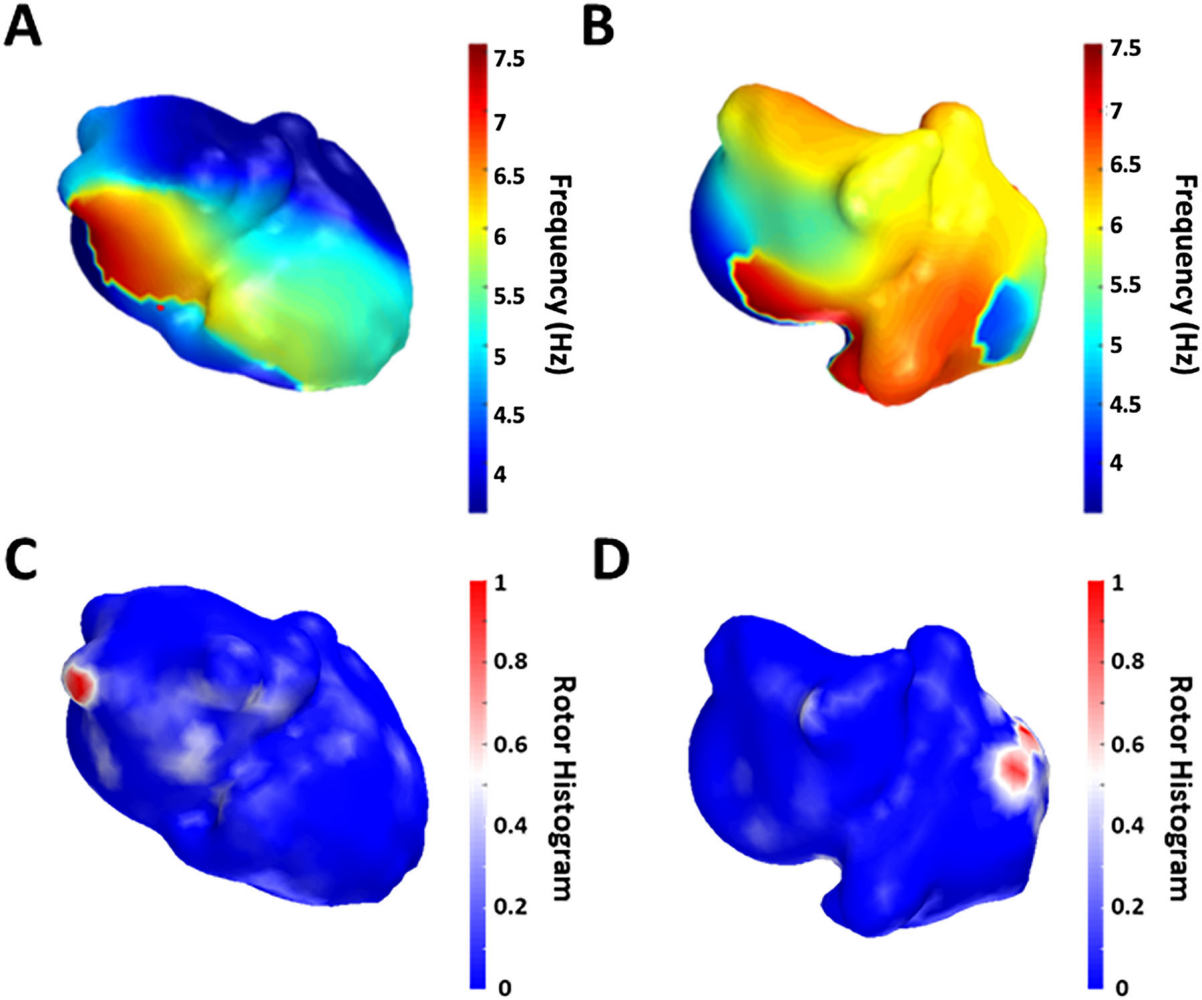
Sample DF and rotor histogram maps from two selected patients. (A) Patient showing simple and homogeneous distribution of activation frequency associated with low values of the ECGI complexity score with localized rotors in the pulmonary vein (panel C). (B) Patient showing a heterogeneous distribution of activation frequency associated with high values of the complexity score showing rotational activity in the right atrium (panel D).

For each patient, ≥ 20 s of AF signals were recorded. QRS complexes in ECG recordings were first removed, and recorded signals from the body surface were filtered with a high pass (Fc = 2 Hz) and a low pass filter (Fc = 20 Hz) [3, 4, 10, 11, 12]. Segments of ≥ 4 s were selected to calculate dominant frequency (DF) maps and 1 s segments were used to calculate rotor maps. This process was repeated for three different time windows per patient, and the results were averaged. Inverse computed electrograms (icEGMs) were calculated using BEM formulation and zero‐order Tikhonov regularization using preselected atrial anatomical models [11, 13]. These parameters were obtained from signals acquired at inclusion, and patients were followed for 12 months. For each recorded segment, we obtained two maps, DF and rotor histogram maps (Figure 1). Briefly, DF was defined as the frequency that presented the highest power calculated with a Welch's periodogram to determine the local DFs with a spectral resolution of 0.01 Hz [13]. Rotor location was carried out by identification of singularity points (SP) in the phase map obtained with the Hilbert Transform [12, 14]. Phase values were obtained along three different circles surrounding each evaluated point, and 6–12 points per circle were used for the phase analysis in which the signal was interpolated by a weighted average of the neighbouring nodes, *d*2 being the weight for each node and *d* the distance between the nodes [12]. An evaluated point was defined as a SP only when the phases of at least two of these three circles was monotonically increasing or decreasing for a total of 2π. A rotor was defined as the connection between SPs across time. The distance between SPs at consecutive time instants should be less than 1 cm (EGM and icEGM) or 5 cm (ECG) to be related and maintain a continuity of rotation. Finally, only long‐lasting rotors, defined as those that complete at least one rotation, were considered as rotors and other SPs were discarded. The complete list of calculated biomarkers can be consulted in Supporting Information S1: Table S1.

### 2.4 Outcome Predictors Evaluation

**Figure 2:**
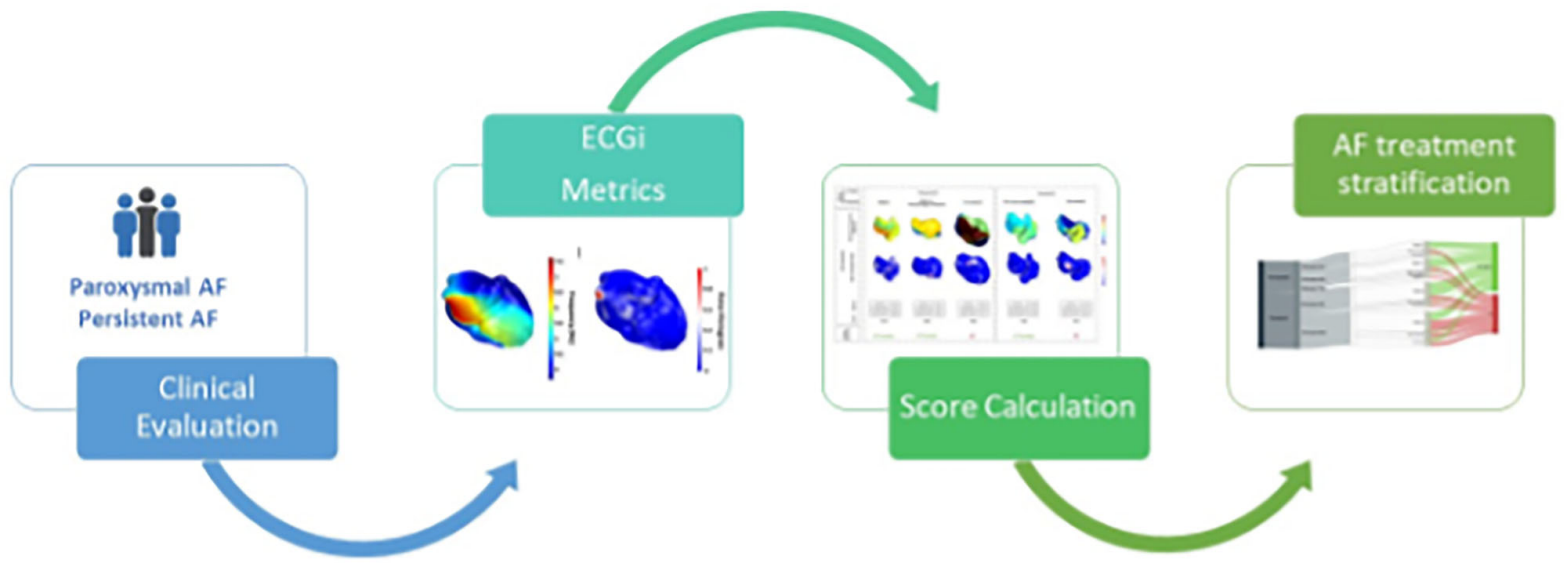
Evaluation of long‐term treatment efficacy (freedom from AF at 1 year) of patients with AF (from left to right): 1. Clinical predictors of treatment efficacy at 1 year using multivariant analysis; 2. Complexity Score Evaluation: ECGI using feature extraction; 3. Score calculation (Formula1); 4: AF treatment stratification: Clustering algorithm analysis.

Statistical tests and analyses were performed once all patients were included, and the endpoint was reached. For all analyses, 80% of patients were included in the training set of the algorithm, and 20% were included in the testing set. The training and test groups preserved the same distribution as the initial pool, to avoid bias in the results. The algorithm was designed to evaluate long‐term treatment efficacy (freedom from AF at 1 year) of patients with AF as follows (Figure 2):

1. Clinical variables: Clinical predictors of treatment efficacy at 1 year were identified using multivariant analysis of clinical data.
2, 3. Complexity score evaluation and score calculation.

Variables obtained from the ECGI were evaluated using feature extraction to identify the most important for 12‐month rhythm outcome evaluation [15]. The 12 variables obtained from the ECGi were evaluated using feature extraction to identify the most important variables for 12‐month rhythm outcome evaluation. The calibration of the ECGi complexity score was calculated using a logistic regression that used three parameters for the decision: Highest Dominant Frequency (HDF), Median Dominant Frequency (Median DF), and Mean Rotor Time.

Formula 1

$$P=\frac{1}{1+e^{-\left(-0.11+0.40*\mathrm{HDF}-0.39*\mathrm{Median}\;\mathrm{DF}-0.04*\mathrm{Mean}\mathrm{Rotor}\mathrm{Time}\right)}}$$

This formula indicates that higher values of HDF increase the score, suggesting higher AF complexity. Conversely, higher values of Median DF decrease the score, indicating lower AF complexity. Shorter Mean Rotor Time also results in a higher score, reflecting greater AF complexity.

4. Clustering algorithm analysis: We assessed if the ECGI complexity score in combination with classical clinical characterization was able to predict the rhythm at 1 year after the inclusion in the study. For this purpose, a clustering algorithm was built to identify profiles that presented similar electrophysiological properties. Briefly, clustering algorithms identify patterns in data with similar baseline characteristics (such as AF type and electrophysiological profile) and similar outcomes (AF or AF freedom at 1 year). The clustering algorithm used in this study consisted of a *k*‐Nearest Neighbour algorithm (*k *= 5) in which three different variables were considered, previously identified in a multivariate analysis: AF type, complexity score, and the type of treatment. Proportions of AF/AF Freedom were similar in both partitions. To define the number of clusters, the following parameters were considered in the analysis, including sample population distribution, minimum number of patients per cluster, and maximum number of combinations based on the final variables included for the training.

### 2.5 Statistical Methods

The Student's *t*‐test was used to evaluate the statistical significance between continuous paired or unpaired variables, and *Z* score was used to calculate *p* values of prevalence (percentages) in the population. Multivariate one‐way analysis of variance was used for multivariate analysis [16, 17, 18]. Statistical significance was considered for *p* < 0.05 for continuous or percentage variables. Pearson *χ* ^2^ Independence test was used to evaluate categorical or binary variables, including comparison of the groups in each of the clusters, and statistical significance was considered for *p* < 0.05. DeLong AUC approach was applied to evaluate the statistical significance of ROC curves [19]. All data are reported as mean ± SD or frequencies and percentages. All ECGI calculations were performed in MATLAB, and training of both the neural networks and the regression algorithm was programmed in Python.

## 3 Results

### 3.1 Patient Cohort Clinical Description: Clinical Variables

We included 204 patients from the outpatient clinic (*N *= 84) and ablation (*N *= 120) groups according to their consecutive referral to each group. Table 1 shows univariate and multivariant clinical predictors of long‐term outcome. Analysis per group (outpatient clinic and ablation) in relation to the outcome can be consulted in Supporting Information S1: Tables S2, S3. Multivariate analysis revealed that both the AF type and treatment strategy were significantly associated with the endpoint (*p* < 0.001), suggesting that these variables should be included in the final model.

**Table 1:** Univariate and multivariant clinical predictors of long‐term outcome.

	All patients	AF Freedom	AF	Univariate analysis	Multivariate analysis	
	204	113	91	*p* value	Confidence interval	*p* value
Gender (female)	74 (36%)	45 (40%)	29 (32%)	0.242	[45–60%]	0.12
Age (years)	60.7 ± 9.4	61.4 ± 10.3	64.9 ± 8.2	0.35	[58.5–65.2]	0.09
Comorbidities						
Arterial hypertension	74 (36%)	41 (36%)	33 (36%)	0.99		
Obesity	24 (12%)	13 (11%)	11 (12%)	0.90		
Ischemic heart disease	3 (1.5%)	3 (1.5%)	0 (0%)	0.12		
Heart failure	5 (2.4%)	3 (1.5%)	2 (2.2%)	0.83		
Mitral regurgitation	5 (2.4%)	3 (1.5%)	2 (2.2%)	0.83		
						
AF type						
Paroxysmal	75 (37%)	47 (42%)	28 (37%)	0.11	[55.0–70.0%]	< 0.01
Persistent	125 (61%)	64 (57%)	61 (37%)			
Treatment strategy						
Rhythm control	137 (67%)	88 (78%)	49 (54%)	< 0.001	[60.0–70.0%]	< 0.05
Rate control	67 (33%)	24 (21%)	43 (47%)			

### 3.2 ECGI Complexity Score Calculation

**Figure 3:**
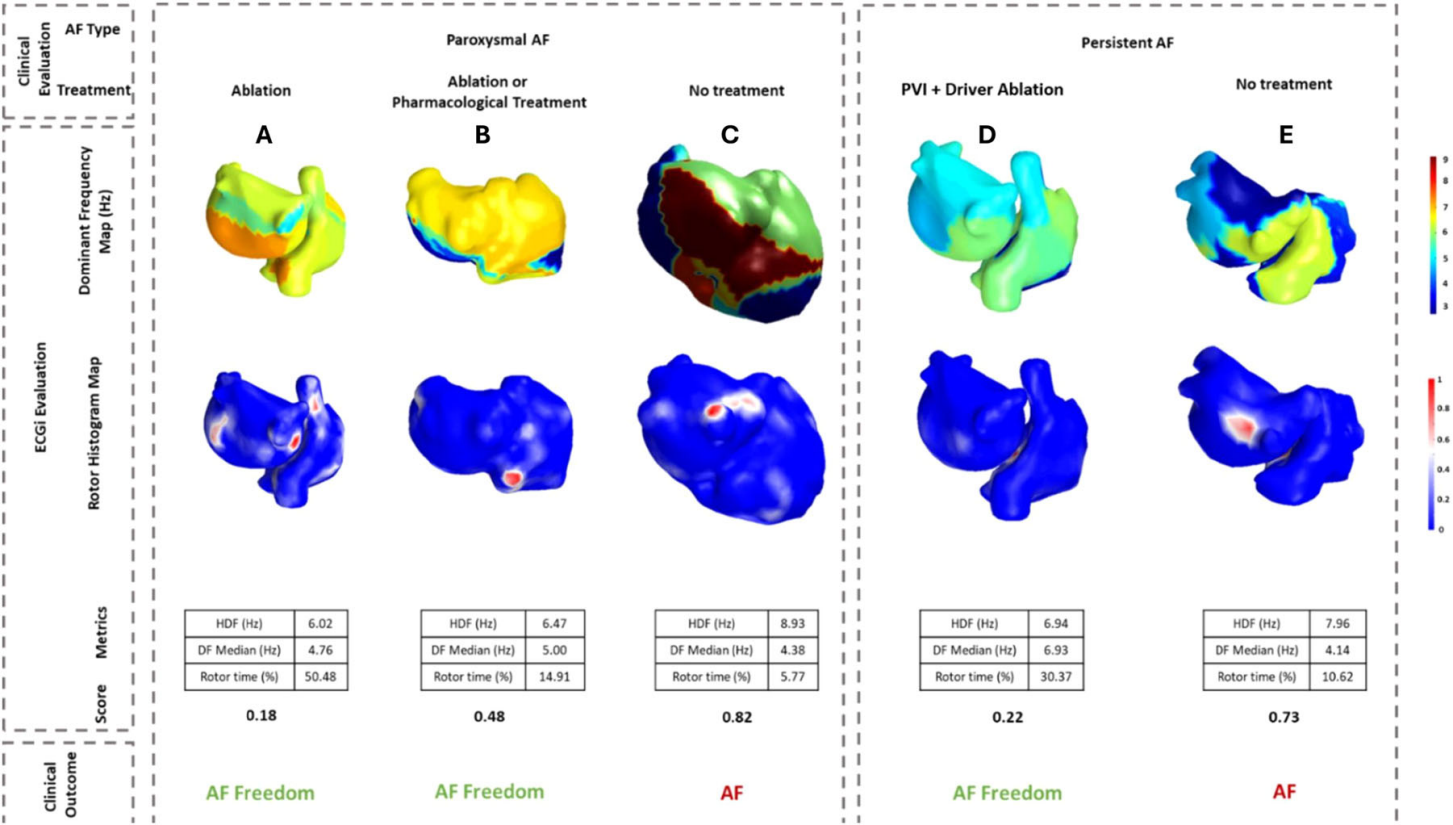
Score calculation: Examples of patients belonging to each of the five clusters. Panel describes the AF type, treatment, dominant frequency map, ECGI biomarkers for each specific patient, calculated score, and clinical outcome.

ECGI metrics were obtained and the ECGI complexity score was calculated. As shown in Figure 3, lower complexity scores presented lower values of HDF, higher median DF values and longer rotor duration time (Figure 3 A,B,D). In contrast, higher values of HDF, lower values of Median DF, and shorter Mean Rotor Time reflected greater AF complexity (Figure 3 C,E).

### 3.3 Global Model for Prediction of Long‐Term Sinus Rhythm Maintenance by Clusters

**Figure 4:**
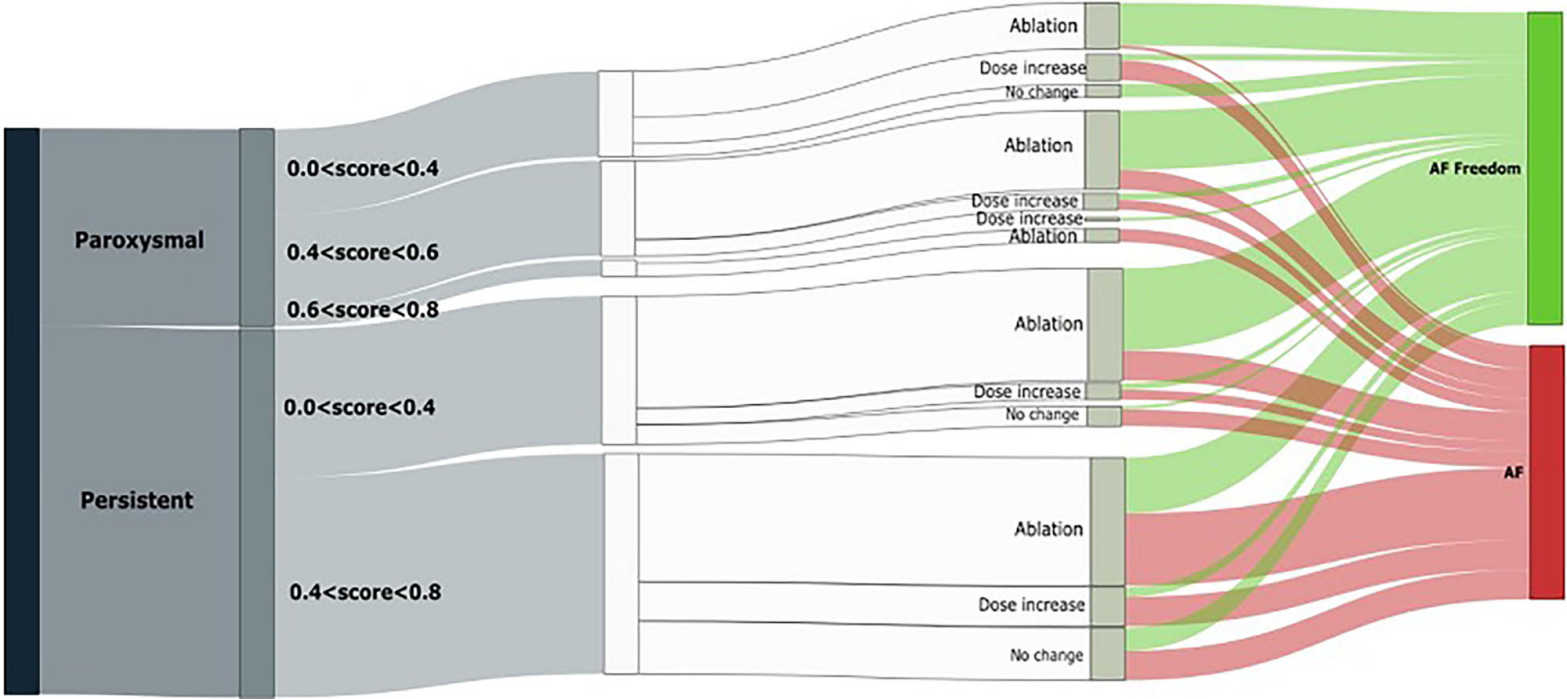
Results obtained from the clustering algorithm identifying 5 different groups in three levels. From left to right, the first variable represents AF type (Paroxysmal on top, Persistent on the bottom), score value divided in three subgroups for the paroxysmal patients and in two groups for the persistent patients and type of treatment and 1‐year outcome in the right part of the diagram. Green and red colours represent AF Freedom and AF at 1 year, respectively.

Three different variables were identified in the multivariate analysis as relevant to predict the outcome of the patients: AF type (paroxysmal vs. persistent AF), ECGI electrophysiological complexity score, and treatment strategy (rate vs. rhythm control), resulting in five differentiated subgroups (Figure 4; Supporting Information S1: image S1). Successful treatments (freedom from AF at 1 year) are shown in green, while the groups showing red colour correspond to unsuccessful treatments (AF at 1 year).

The first group, corresponding to paroxysmal patients with low complexity score values, presents better outcomes (SR) when ablation was performed (AF freedom at 1‐year: ablation group: 93% vs. pharmacological treatment: 25%; *p* value: 0.014). The second group, corresponding to paroxysmal patients with intermediate score values between 0.4 and 0.6, presented a trend for higher probability of SR maintenance with ablation (AF freedom at 1‐year: ablation: 75% vs. pharmacological treatment: 40%; *p* value: 0.12) and also showed higher probability of SR at 1‐year using rhythm control treatment strategies (rhythm control treatments 85% vs. frequency control treatment 15%; *p* value: 0.002). The third group, corresponding to paroxysmal patients with high complexity score values, presented a lower probability of SR maintenance at 1 year independently of the treatment strategy used (no statistical tests could be applied due to the reduced number of patients (*n* = 4) in this cluster.

In the case of persistent AF patients, those with a low complexity score value presented a trend for higher probability of SR with ablation (AF freedom at 1‐year: ablation: 62% vs. pharmacological treatment: 40%; *p* value: 0.1) at 1 year. Moreover, in this group, the ablation strategy of driver ablation combined with PVI increased the probability of SR at 1 year (PVI + drivers ablation: 75% (16 patients) vs. PVI: 25% (5 patients); *p* value: 0.005). Finally, the fifth group, corresponding to persistent patients with high score values, showed an overall poor outcome regardless of the type of treatment (AF freedom at 1 year: ablation: 44% vs. pharmacological treatment: 25% vs. others: 44%; *p* value: 0.52).

Figure 3 shows one example for each of the clusters and the calculated ECGI complexity metrics for each group. Low electrophysiological complexity patterns were associated with an improved outcome after ablation, regardless of the time duration of the AF, demonstrating the importance of including the AF complexity values. Interestingly, for paroxysmal patients with a score between 0.4 and 0.6, rhythm control treatments can be effective, while in the case of the same patient being persistent, the outcome is not very favourable. In contrast, cluster patterns with higher electrophysiological complexity were associated with a higher probability of AF recurrence, both in paroxysmal and persistent groups.

Finally, this algorithm was evaluated on the 20% test set, obtaining 90% prediction success, showing better performance than the conventional paroxysmal versus persistent classification (*p *< 0.05). The AUCs for each of the models (Table 2), demonstrate an increase in prediction performance using the ECGI Complexity Score as compared to the temporal pattern‐based classification of AF.

**Table 2:** Receiver operator characteristic curves for prediction models of patients using the ECGI complexity score and the 3‐P classification score.

Type of score	AUC	Sensitivity	Specificity	Positive predictive value	Negative predictive value	*p* value
ECGI complexity score	0.73 (0.63–0.81)	80.00% (68.23%–88.90%)	57.14% (42.21%–71.18%)	71.23% (63.68%–77.77%	68.29% (55.57%–78.76%)	*p* < 0.05
3‐P classification	0.58 (0.48–0.68)	72.73% (57.21%– 85.04%)	32.00% (21.69%–43.78%)	38.55% (33.08%– 44.33%)	66.67% (52.71%–78.21%)	

Abbreviations: AUC, area under the curve; 3‐P classification, temporal pattern‐based classification score.

## 4 Discussion

The major findings of the present study are as follows. First, ECGI enabled noninvasive, simultaneous, global characterization of bi‐atrial electrical activity. Second, the evaluation of AF electrophysiological complexity combined with the clinical information increased the performance of conventional outcome prediction methods for AF treatment selection. Finally, an AI‐driven algorithm enabled AF treatment stratification in a nonselected population based on noninvasive AF complexity analyses.

### 4.1 AF Treatment Stratification

Antiarrhythmic drug therapy is the cornerstone of AF clinical management, doubling the probability of maintaining sinus rhythm compared with rate‐control therapy [20]. Moreover, the EAST‐AFNET 4 trial found that early comprehensive rhythm control reduced the risk of adverse cardiovascular outcomes versus usual care, where antiarrhythmic drugs remain an important treatment option for most patients with AF [21]. However, their use has been conditioned by drug toxicity effects and interactions with concomitant cardiovascular conditions, reducing the likelihood of long‐term drug tolerance. Progress in AF ablation techniques showed an incremental benefit compared with pharmacological interventions. Despite the results of randomized rhythm versus rate control trials showing no survival advantage, recent studies demonstrated an increase in long‐term outcomes using ablation therapies in selected subgroups [22]. Although rate control is the initial default treatment in current guidelines, AF treatment pattern is moving towards offering rhythm control strategies to a wider population [1, 2, 22]. However, < 25% of patients with AF are treated with rhythm‐control therapy in general AF registries, due to the limited efficacy of drug‐based treatments [1, 23]. Moreover, the opportunity to apply an invasive rhythm control strategy is limited by the reduced efficacy in subgroups of patients, safety concerns, and availability of ablation resources for the overall population of AF patients. Finally, rate control may also be needed as an end‐stage strategy for many patients with AF, from asymptomatic patients to patients with failed drug or interventional treatments. Therefore, stratification methods are critical to accurately select treatment strategies for the overall population of patients with AF.

The temporal pattern‐based classification of AF (3‐P classification) provided a simple and easily adopted classification of the disease but lacks precision in differentiating among optimal treatment decisions, such as rhythm versus rate control strategies [24]. This heterogeneous system describes only the arrhythmia temporal behaviour, whereas other relevant features, such as cardiovascular risk factors or the impact of atrial remodelling, were not considered. In our study, the 3‐P classification failed to demonstrate an association with cardiovascular outcomes.

### 4.2 Role of Noninvasive AF Complexity Evaluation

The 4S‐AF structured pathophysiology‐based characterization scheme, addressing specific domains with treatment and prognostic implications, has been recently proposed and adopted by the ESC Guidelines [1, 25]. Consequently, other variables such as circulating biomarkers, ECG‐based parameters, imaging, and individual genetic profile are currently being incorporated for a more robust evaluation of candidate predictors for recurrent AF after ablation in studies under development [26]. Recent developments of ECGI systems that enable noninvasive, simultaneous, global characterization of bi‐atrial electrical activity have been associated with AF termination during ablation [2, 10]. Moreover, the recently developed imageless ECGI eliminates the need for CT or MRI by estimating cardiac geometry and location based on electrical, statistical, and thoracic geometrical data, significantly reducing costs and scan times [3].

Following this approach, we analyzed the electrophysiological complexity using ECGI, in combination with clinical and substrate characteristics, to predict long‐term treatment efficacy (freedom from AF at 1 year) of an unselected population of patients. To that purpose, we developed a new stratification score based on previously identified biomarkers of ablation efficacy using ECGI recordings [6, 9, 10, 13, 27]. This new score allowed the identification of five different clusters that increased the performance of conventional clinical guidelines for treatment selection. Although the sizes of prior studies were larger, none of them considered AF across all clinical labels nor different treatment approaches besides ablation [16, 17, 18]. In contrast, we used non‐invasive electrophysiological parameters obtained from the ECGI combined with clinical data to predict the outcome in the overall population of patients with AF.

Our risk score includes several parameters that have already been related to AF prediction, such as the highest dominant frequency and the rotor time [5, 13, 28]. In particular, HDF has been previously identified as one of the biomarkers for efficacy evaluation of patients undergoing ablation using the ECGI technology [6, 13, 14, 28]. For paroxysmal AF patients, ablation will be the ideal intervention for patients with a low complexity score; the intermediate score group could receive rhythm treatments using either drugs or ablation, while patients with the highest complexity could be considered for more aggressive ablation strategies (including AF surgery) or rate control strategy. In the case of persistent patients, those in the lower complexity cluster may benefit from rhythm control strategies, using either drugs and/or ablation, while personalized ablation or rate control strategy could be considered in patients in the highest complexity group. However, a non‐negligible proportion of patients in the high complexity persistent AF cluster may still benefit from ablation, but the long‐term benefits of ablation are low. Consequently, we propose a new workflow for the overall population of AF patients, allowing the identification of the most effective treatment strategy (personalized) that may increase the probability of resulting in sinus rhythm maintenance at 1 year (Figure 2).

Prior studies have evaluated the electrical complexity of AF, demonstrating that most paroxysmal AF drivers are located close to the PV antrum, while persistent AF patients have a more evenly spaced distribution of drivers outside the PV antra to the body of the atria [10, 27, 28, 29]. Lim et al. found that the complexity of AF drivers increased with longer AF duration, but these results were restricted to persistent AF patients [28]. Rodrigo et al. showed that the number of reentrant activity regions was inversely related to the severity of AF across clinical labels of AF and was related to the probability of acute termination of AF during the procedure [10]. Arrhythmia complexity of body surface recordings has also been characterized using time‐domain parameters. Lankveld et al. identified the mean fibrillation‐wave amplitude as an independent predictor of long‐term persistent AF prognosis [30]. Similarly, Matsuo et al. found that surface ECG AF cycle length independently predicts procedural termination and outcome after long‐lasting persistent AF ablation [31]. In another study, Meo et al. [32] proposed a noninvasive analysis of body surface potential mapping signals and described complex arrhythmias as short cycle lengths and higher nondipolar component index. Overall, these studies suggest that the complexity of AF recordings as shown by the increase in dominant frequency and increase in number of reentrant patterns with a widespread location in both atria, reduce the likelihood of SR maintenance with drugs or current ablation therapies.

### 4.3 Role of AI Driven Analysis

There is little data about the best treatment approach for a given patient at the time of AF. Whereas classical clinical predictors are particularly limited for this purpose, AI techniques are promising to predict AF outcome [8, 15, 33, 34]. Here, we combined classical multivariant breakdown data with cluster analysis to measure differences between individual cases to find clusters of patients with similar outcomes. First, temporal pattern‐based classification of AF initially drove cluster formation. Second, ECGI complexity determined the identification of five clinically relevant clusters. Finally, these distinct clusters were associated with different treatment patterns, including rhythm control and/or rate control treatment strategies.

Inohara et al. used cluster classification to identify clinically relevant categories and phenotypes of patients with AF that were associated with different risks for major cardiovascular and neurologic adverse events [33]. Other studies applied multilayer techniques to predict recurrence following drug treatment and ablation by combining clinical data with imaging and/or electrogram recordings after AF ablation [34, 35, 36]. Shade et al. utilized a machine learning model of clinical data with magnetic resonance imaging scans that increased the predictability of the model [34]. Kim et al. performed clinical decision AI analyses to identify rhythm‐control potential candidates, at the expense of not knowing which specific predictors were used by the model [35]. More recently, the development of a multimodal fusion of intracardiac electrograms, 12‐lead ECG, and clinical features provided the highest performance in predicting 1‐year AF outcomes after ablation [36]. Although the clinical utility of these specific AF phenotypes should be validated in independent populations, they may improve the risk assessment of AF recurrence and guide therapy.

### 4.4 Limitations

The present work has several limitations. The cohort was recruited in a single hospital and a wider sample from different hospitals with external validation will ensure robustness of the risk score. In addition, the cohort presented imbalanced data sets, with the ablation subgroup including more patients. As the difference was not significantly high, we assumed that balancing group methods were not necessary for the development of the score, but further studies should confirm this as well as a validation of the proposed clusters, that should be confirmed to ensure that the methodology and the sample used for training are representative of the population in other clinical scenarios (different hospitals and ethnic populations). Secondly, despite the absence of a predefined treatment at inclusion, clustering algorithm results were associated with different outcome patterns. Nevertheless, a non‐negligible proportion of patients in the rate control group were in SR at the end of follow‐up. Thirdly, AF complexity could only be analysed in patients presenting AF during the outpatient visit or during the electrophysiologic procedure, potentially excluding patients with lower AF burden. However, we are currently aiming to analyse these features during sinus rhythm in ongoing protocols [37]. Finally, a larger sample of patients should be analyzed to further investigate the role of the antiarrhythmic treatment.

## 5 Conclusions

Patient evaluation and stratification before treatment decision is key to improving the success rate of current AF patient management. AI‐driven analysis that combined clinical information with electrophysiological ECGI biomarkers increased the performance of conventional classification methods for AF treatment stratification.

## Members of the STRATIFY Study Group

Francisco Fernández‐Avilés, MD, PhD; Pablo Ávila, MD, PhD; Tomás Datino, MD, PhD; Nina Soto, MD; Paloma Pérez‐Espejo, MD; Francisco Cruz, MD; Valentina Garello, MD; Roberto Gómez, MD; Ines Martin, MSc; and Laura González, MSc from the Department of Cardiology, Hospital General Universitario Gregorio Marañón, Instituto de Investigación Sanitaria Gregorio Marañón, Madrid, Spain, and CIBERCV, Centro de Investigación Biomédica en Red de Enfermedades Cardiovasculares, Spain.

## Ethics Statement

The study protocol was reviewed and approved by the Institutional Review Board.

## Consent

All patients gave written informed consent per IRB guidelines.

## Conflicts of Interest

F.A., F. F‐A., A.M.C., and M.S.G. have equity from Corify Care SL. F.A. served on the advisory board of Medtronic. P.A. received teaching honoraria from Medtronic and served on the advisory board of Boston Scientific.

## Supporting information

SupMaterial.

## Data Availability

The data underlying this article will be shared on reasonable request to the corresponding author.
