# Supplementary material for: Atrial Fibrillation Treatment Stratification Based on Artificial Intelligence‐Driven Analysis of the Electrophysiological Complexity
Source: J Cardiovasc Electrophysiol. 2025 Jun 4;36(8):1903–12. doi: 10.1111/jce.16754 (PMC12337632; doi:10.1111/jce.16754)

**Supplemental Materials**

Table S1. Evaluation metrics obtained from the ECGI

|  | Outcome Measure | Description |
| --- | --- | --- |
| Frequency  Biomarkers | Dominant Frequency (Hz) | Highest magnitude sinusoidal component of the Electrogram found by decomposing the electrograms into a finite number of sinusoidal constituents and finding the one with the highest magnitude. |
|  | Highest Dominant Frequency (Hz) | Largest DF value among those estimated for each atrial site. |
|  | Highest Dominant Frequency Extension (%) | Extension, expressed in percentage, of the Highest Dominant Frequency in both atrial cavities. |
|  | Median Dominant Frequency (Hz) | Median DF value among those estimated for each atrial site. |
|  | Median Dominant Frequency Extension (%) | Extension of the median Dominant Frequency calculated from the ECGI signals of both atria. |
|  | Minimum Dominant Frequency (Hz) | Minimum value of the Dominant Frequencies calculated from the ECGI signals of both atria. |
| Rotor  Biomarkers | Rotor per second | Number of identified rotors per second in both atrial cavities. |
|  | Mean simultaneous rotor | Mean number of rotors identified simultaneously occurring in both atrial cavities. |
|  | Mean rotor duration | Mean rotor duration of the identified rotors calculated as a percentage for the selected ECGI segment |
|  | Singularity Points at Highest Dominant Frequency | Singularity points or nodes of the atria that present the highest dominant frequency |
|  | Singularity Points per second | Number of singularity points per second considering both atrial cavities. |
|  | Mean rotor time (%) | Defined as the average of the duration of all identified rotors |
|  | Entropy | Value of the entropy calculated for the ECGI signals. |
|  | Calculated score | Score for the evaluation of the electrophysiological complexity |

Table S2. Univariate predictors of AF freedom of outpatients with AF.

|  | All patients | AF Freedom | AF | p-value |
| --- | --- | --- | --- | --- |
|  | 84 | 33 | 51 |  |
| Gender (female) | 32 (38%) | 18 (56%) | 14 (27%) | 0.99 |
| Age (yrs) | 65.4 ± 11 | 64.7 ± 12.5 | 65.8 ± 10 | 0.67 |
| Comorbidities | | | | |
| Arterial Hypertension | 40 (48%) | 17 (34%) | 23 (45%) | 0.79 |
| Obesity | 9 (11%) | 1 (3%) | 8 (16%) | 0.07 |
| Ischemic Heart disease | 2 (2%) | 2 (6%) | 0 (0%) | 1.00 |
| Heart Failure | 3 (4%) | 1 (3%) | 2 (4%) | 0.66 |
| Mitral Regurgitation | 3 (4%) | 2 (6%) | 1 (2%) | 0.94 |
|  |  |  |  |  |
| AF Type | | | | |
| Paroxysmal | 31 (37%) | 13 (39%) | 18 (35%) | 0.93 |
| Persistent | 53 (63%) | 20 (61%) | 33 (65%) |  |

Table S3. Univariate predictors of AF freedom of patients undergoing AF ablation.

|  | All patients | AF Freedom | AF | p-value |
| --- | --- | --- | --- | --- |
|  | 120 | 80 | 40 |  |
| Gender (female) | 42 (35%) | 27 (34%) | 15 (38%) | 0.417 |
| Age | 60.9 ± 9.3 | 59.1 ± 9.5 | 64.4 ± 7.8 | 0.003 |
| Comorbidities | | | | |
| Arterial Hypertension | 34 (29%) | 24 (30%) | 10 (25%) | 0.78 |
| Obesity | 15 (12%) | 12 (10%) | 3 (0.75%) | 0.93 |
| Ischemic heart disease | 1 (1%) | 1 (1.25%) | 0 (0%) | 1.00 |
| Heart Failure | 2 (1.7%) | 2 (2.5%) | 0 (0%) | 1.00 |
| Mitral Regurgitation | 2 (1.7%) | 1 (1.25%) | 1 (2.5%) | 0.56 |
|  |  |  |  |  |
| Left Atrial Diameter (cm) | 4.14 ± 0.59 | 4.08 ± 0.59 | 4.30 ± 0.57 | 0.07 |
| Left Atrial Area (cm^2^) | 24.36 ± 6.16 | 22.95 ± 5.52 | 27.57 ± 6.46 | 0.001 |
| AF Type | | | | |
| Paroxysmal | 44 (37%) | 33 (41%) | 11 (27%) | 0.14 |
| Persistent | 76 (63%) | 47 (59%) | 29 (72%) |  |
| Ablation type | | | | |
| PVI | 50 (42%) | 37 (46%) | 13 (32%) | 0.15 |
| PVI + drivers | 70 (58%) | 43 (54%) | 27 (67%) |  |

Image S1. Cluster distribution


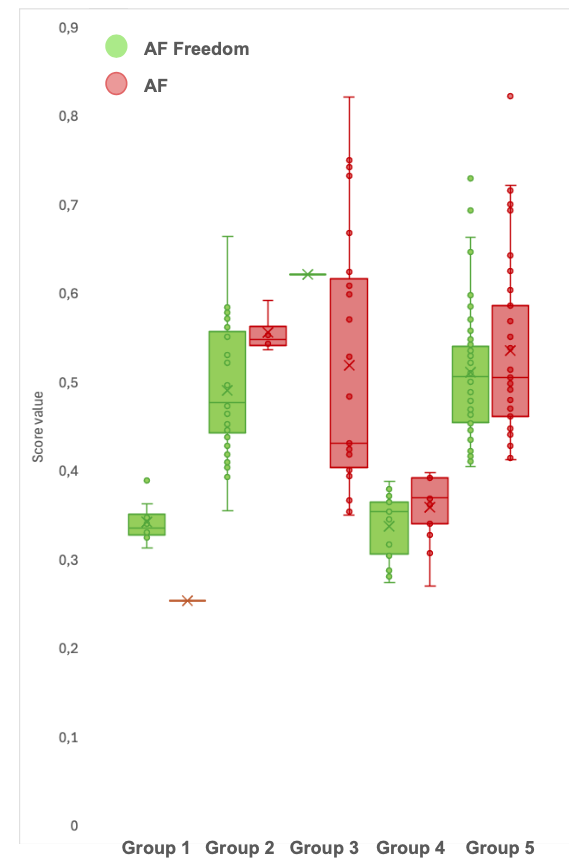

Supplement: Supplementary file 1 — SupMaterial. [file JCE-36-1903-s001.docx]
